# An Information Geometry-Based Track-Before-Detect Algorithm for Range-Azimuth Measurements in Radar Systems †

**DOI:** 10.3390/e27060637

**Published:** 2025-06-14

**Authors:** Jinguo Liu, Hao Wu, Zheng Yang, Xiaoqiang Hua, Yongqiang Cheng

**Affiliations:** College of Electronic Science and Technology, National University of Defense Technology, Changsha 410073, China; liujinguo19@nudt.edu.cn (J.L.); yangzheng18@nudt.edu.cn (Z.Y.); huaxiaoqiang12@nudt.edu.cn (X.H.); yqcheng@nudt.edu.cn (Y.C.)

**Keywords:** multi-frame detection, information geometry, track before detect, dynamic programming, range-azimuth measurements, Kullback–Leibler divergence

## Abstract

The detection of weak moving targets in heterogeneous clutter backgrounds is a significant challenge in radar systems. In this paper, we propose a track-before-detect (TBD) method based on information geometry (IG) theory applied to range-azimuth measurements, which extends the IG detectors to multi-frame detection through inter-frame information integration. The approach capitalizes on the distinctive benefits of the information geometry detection framework in scenarios with strong clutter, while enhancing the integration of information across multiple frames within the TBD approach. Specifically, target and clutter trajectories in multi-frame range-azimuth measurements are modeled on the Hermitian positive definite (HPD) and power spectrum (PS) manifolds. A scoring function based on information geometry, which uses Kullback–Leibler (KL) divergence as a geometric metric, is then devised to assess these motion trajectories. Moreover, this study devises a solution framework employing dynamic programming (DP) with constraints on state transitions, culminating in an integrated merit function. This algorithm identifies target trajectories by maximizing the integrated merit function. Experimental validation using real-recorded sea clutter datasets showcases the effectiveness of the proposed algorithm, yielding a minimum 3 dB enhancement in signal-to-clutter ratio (SCR) compared to traditional approaches.

## 1. Introduction

The detection of weak moving targets in complex scenarios is an important issue in fields such as radar, sonar, and communication [1,2]. Complex scenarios such as airborne down-view scenes, strong clutter, and high maneuverability of targets, cause low signal-to-clutter ratios (SCRs) and power fluctuations, thereby causing target detection misses. In recent years, comprehensive studies have been carried out on the target detection theory [3]. Typically, non-coherent processing methods rely on the energy of the received signal for detection [4]. They have generated interest for many years due to their convenient implementation [5]. In the traditional pulse Doppler (PD) radar system, the moving target detection (MTD) technique (realized by fast Fourier transform (FFT) processing) is usually used in conjunction with the constant false alarm rate (CFAR) detector [6]. Besides these methods discussed earlier, notable approaches such as the generalized likelihood ratio test (GLRT) detector [7,8], Rao, and Wald tests [9,10] depend on covariance matrix estimation. The precision of the clutter covariance matrix (CCM) estimation is vital for ensuring better detection performance [11]. However, the availability of homogeneous clutter samples for CCM estimation is limited, which significantly degrades the performance [12].

Over the past several years, a novel radar detection technique based on information geometry (IG) theory has been rapidly developed [13,14,15,16]. The IG detector demonstrates significant performance advantages in signal detection problems under limited sample and heterogeneous clutter, avoiding the issue of low Doppler resolution caused by short pulse conditions [17,18]. The detector that uses covariance matrices to characterize the observed data is known as a matrix information geometry (MIG) detector [19,20]. This idea is to consider each covariance matrix as a point on the Hermitian positive definite (HPD) manifold. It characterizes the dissimilarity between the target and the clutter by measuring the geometric distance between the cell under test (CUT) and reference cells on the manifold [21]. The Riemannian distance is a metric that was initially employed [13]. This method has shown better performance in X-band and high-frequency ground-wave radar target detection [22]. However, information divergence has been proposed for geometric metrics on manifolds due to the high computational complexity of the Riemannian metric, with the Kullback–Leibler (KL) divergence being the most commonly used [15]. In addition to the covariance matrix, other distinguishable correlation characteristics can be utilized to construct the manifold space, such as the power spectrum (PS) [23] and the correlation feature (CF) [16]. However, single-frame processing on the manifold also lacks sufficient information for weak target detection in strong clutter backgrounds. Hence, the multi-frame processing strategy needs to be considered.

As a reliable multi-frame processing strategy, track-before-detect (TBD) technology has emerged [24,25]. The fundamental principle underlying TBD is to suppress clutter while fully leveraging the spatiotemporal correlation of the target to integrate target energy or test statistics. Common methods for TBD include the Hough transform [26], velocity filtering [27], particle filtering [28,29], and dynamic programming (DP) [30]. Currently, TBD methods are primarily designed for specific scenarios [31,32,33,34]. For instance, to address the radar field-of-view misalignment issue, a multi-frame energy integration strategy for moving platform radars has been proposed [34]. A TBD method with soft orbit-information constraints is proposed to address the problem of detecting distant or dim space targets in space target surveillance [35]. The aforementioned methods have introduced advanced approaches for scenarios such as misaligned automotive fields of view, distant, or dim space targets, which can improve detection performance in the corresponding scenarios. However, these methods are still based on energy integration, which remains a formidable challenge in the context of weak target detection [36]. In contrast, the IG approach integrates multidimensional information about the target across frames, transcending the limitations of mere energy integration. This endows it with the unique advantage of detecting weak targets. Existing IG-based multi-frame detection methods are mainly focused on staring scenarios [33,37]. Nevertheless, in application scenarios, such as coastal surveillance radars, early warning radars usually adopt the scanning mode to obtain range-azimuth measurements [38]. Due to the long frame intervals in the azimuth scanning mode, multi-frame processing of range-azimuth measurements must account for the target’s motion model to accurately predict the target’s position, which is different from staring mode radars [39]. Hence, it is necessary to develop multi-frame detection schemes suitable for range-azimuth measurements.

To tackle the challenges outlined above, this work introduces an IG-based DP-TBD algorithm for range-azimuth measurements. The main contributions are outlined as follows:

- **IG-based inter-frame information integration for range-azimuth measurements:** During the time interval between adjacent frames, the motion of the target induces changes in the features on the manifold. By analyzing the geometric properties of the target track and clutter track on the manifold, the information from range-azimuth measurements is integrated across multiple frames. Within the framework of information geometry detection, the target and clutter maintain distinguishability under multi-frame conditions. Meanwhile, the similarity between targets across frames introduces new information for integration.
- **IG-based DP-TBD algorithm for range-azimuth measurements:** Utilizing the distinctive features of targets and clutter on the manifold, a scoring function based on manifold distances is designed within the IG framework. An IG-based DP-TBD algorithm is derived to extract target trajectories that have the maximum integrated merit function values.
- **Numerical experimental validation of real-recorded sea clutter data:** Experiments using sea clutter data were performed to substantiate the effectiveness of the proposed methods. The findings confirm that these methods can accurately estimate target trajectories. Moreover, the detection performance of the proposed method provides at least a 3 dB improvement in SCR compared to traditional methods.

The rest of this article: Section 2 formulates the problem and preliminaries of IG detector. The IG-based DP-TBD algorithm for range-azimuth measurements is introduced in Section 3. Experiments and analysis are proposed in Section 4. Finally, Section 5 briefly draws the conclusion.

## 2. Problem Formulation and Preliminaries of IG Detector

This section will first introduce the signal model and detection model. Then, the measurement model and multi-frame detection scheme for range-azimuth measurements is formulated. Subsequently, the IG detector under single-frame detection is briefly described.

### 2.1. Signal Model and Detection Model

An echo measurement sensed by a radar sensor is examined, which divides the observation space into $N_{r}$ range cells and receives *N* pulse samples from each cell. The measurement received from the *i*-th range cell is a *N*-dimensional complex vector, i.e., $z_{i}\in C^{N\times 1},\;i=1,2,\ldots ,N_{r}$, where C denotes the set of complex numbers. The echo signal for each range cell within a coherent processing interval (CPI) can be represented as an *N*-dimensional complex vector $z={\left[z_{0},z_{1},\ldots ,z_{N-1}\right]}^{T}$. We model the radar target detection problem as a binary hypothesis testing problem.

(1) $$\left\{\begin{array}{cc} \; & H_{0}:\left\{\begin{array}{cc} \; & z_{D}=c\\ \; & z_{k}=c_{k},\;k=1,2,\ldots ,N_{R}, \end{array}\right.\\ \; & H_{1}:\left\{\begin{array}{cc} \; & z_{D}=As+c,\\ \; & z_{k}=c_{k},\;k=1,2,\ldots ,N_{R}, \end{array}\right. \end{array}\right.$$

where $z_{D}$ represents the data obtained from the CUT, $z_{k}$ corresponds to the data obtained from the *k*-th reference cell, $N_{R}$ indicates the total number of reference cells, and $c_{k}$ and c are the clutter data. The target signal can be represented as p=As, where *A* is the amplitude parameter and s is the target steering vector.

(2) $$s=\frac{1}{\sqrt{N}}{\left[1,\exp\left(-j2\pi f_{d}\right),\ldots ,\exp\left(-j2\pi f_{d}\left(N-1\right)\right)\right]}^{T}$$

where $f_{d}$ denotes the normalized Doppler frequency and *N* is the total number of pulses within a CPI.

**Figure 1:**
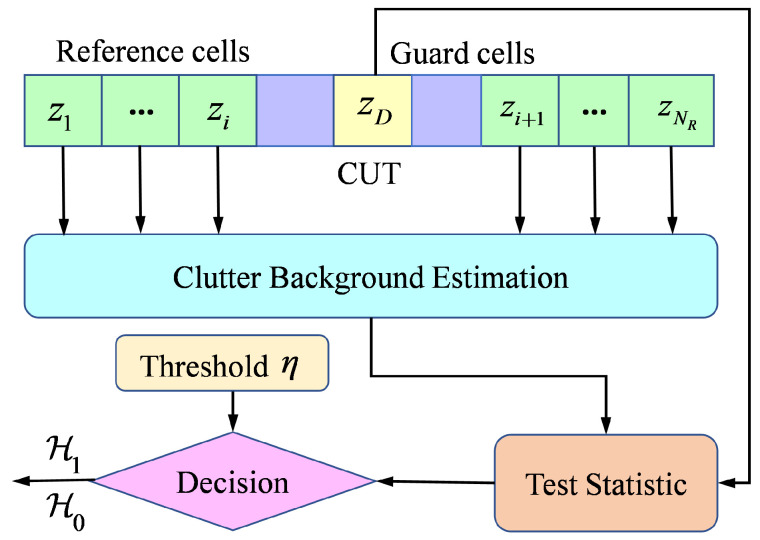
Detection scheme in a coherent processing interval.

The detection process for a CPI in radar signal processing is clearly illustrated in Figure 1. Firstly, the system estimates the clutter background using the data from the reference cells. Next, a threshold value is set based on the estimated background. Then, the test statistic is calculated and compared with the threshold. If it surpasses the threshold, it is identified as a detected target; otherwise, it is deemed to indicate the absence of a target.

When a radar continuously scans an observation area, the target echoes often fall into different range cells within a CPI due to the target’s motion. Consequently, the inefficient integration of target energy leads to degraded detection performance, particularly in cases where the target echo power is reduced. To tackle the issue, incorporating inter-frame information from multi-frame scanning data can significantly improve performance. The following section will introduce the inter-frame integration process for multi-frame information.

### 2.2. Measurement Model and Multi-Frame Detection Scheme

**Figure 2:**
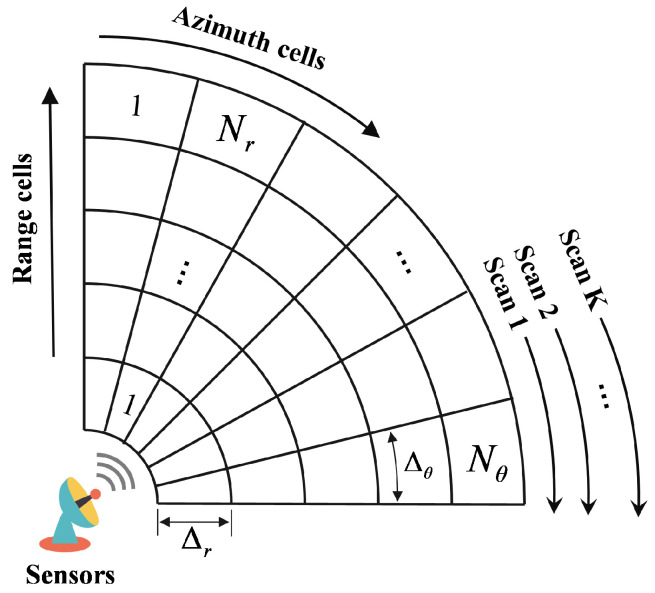
Range-azimuth measurements of radar systems.

A radar system divides the range-azimuth plane into $N_{r}\times N_{\theta }$ resolution cells of size $\Delta_{r}\times \Delta_{\theta }$, as illustrated in Figure 2. Operating with scanning period *T*, it collects measurement data at frames k=1,…,K. Notably, while target motion is typically characterized in Cartesian coordinates, the radar actually receives echo in polar coordinates with the radar as a point of reference.

Assuming that *K* frames of echo data are processed simultaneously during a multi-frame processing, the target discrete grid state at the *k*-th frame can be expressed as

(3) $$x_{k}={\left[\begin{array}{c} r_{k},{\dot{r}}_{k},\theta_{k},{\dot{\theta }}_{k} \end{array}\right]}^{T}\in R^{4}$$

where $r_{k}$ and $\theta_{k}$ represent the position coordinates, and ${\dot{r}}_{k}$ and ${\dot{\theta }}_{k}$ represent the velocity coordinates in the range and azimuth dimensions. The size of set $R^{4}$ satisfies

(4) $$\left|R^{4}\right|=N_{r}\times N_{\dot{r}}\times N_{\theta }\times \left\lceil \frac{\left({\dot{\theta }}_{\max}-{\dot{\theta }}_{\min}\right)}{\Delta_{\dot{\theta }}}\right\rceil$$

where ${\dot{\theta }}_{\max}$ and ${\dot{\theta }}_{\min}$ represent the upper and lower bounds of the azimuth velocity, $N_{\dot{r}}$ represents the length of Doppler cells, and where the azimuth velocity resolution cell size is given by $\Delta_{\dot{\theta }}=\Delta_{\theta }/T$.

Under the constant velocity (CV) motion model, the target’s state transition can be described below,

(5) $$x_{k}=Fx_{k-1}+\Gamma v_{k-1}$$

with

(6) $$F=I_{2}⊗\left[\begin{array}{cc} 1 & T\\ 0 & 1 \end{array}\right],\;\Gamma =I_{2}⊗\left[\begin{array}{c} \frac{T^{2}}{2}\\ T \end{array}\right],$$

where $I_{2}$ represents a 2×2 identity matrix and ⨂ is the Kronecker product. Since a white Gaussian process noise sequence is assumed,

(7) $$v_{k-1}={\left[\begin{array}{c} v_{r},v_{\theta } \end{array}\right]}^{T}∼N\left(0,Q\right)$$

Here, $v_{r}$ and $v_{\theta }$ represent the noisy “acceleration” terms in the r and θ directions, respectively. Under these conditions, the covariance matrix of the process noise can be formulated as

(8) $$Q=\left[\begin{array}{cc} \mathrm{var}(v_{r}) & 0\\ 0 & \mathrm{var}(v_{\theta }) \end{array}\right]=\left[\begin{array}{cc} \sigma_{r}^{2} & 0\\ 0 & \sigma_{\theta }^{2} \end{array}\right]$$

where $\sigma_{r}^{2}$ and $\sigma_{\theta }^{2}$ denote the noise variance in the r and θ directions.

In addition to this model, there are cooperative turning (CT) and constant acceleration (CA) models. The corresponding motion models can be implemented by modifying the parameters [40]. This will not be repeated here.

Thus, the measurements are denoted as $Z_{1:K}=\left\{Z_{1},Z_{2},\ldots ,Z_{K}\right\}$. Each frame is measured as a two-dimensional plane of pixel points.

(9) $$Z_{k}=\left\{z_{k}^{r,\theta }\right\},\;r=1,\ldots ,N_{r},\;\theta =1,\ldots ,N_{\theta }$$

Building upon the aforementioned model, multi-frame integration detection can be articulated as

(10) $$\left\{\begin{array}{c} H_{0}:z_{k}^{r,\theta }=w_{k}^{r,\theta },\\ H_{1}:z_{k}^{r,\theta }=\left\{\begin{array}{cc} A_{k}s_{k}+w_{k}^{r^{\prime },\theta^{\prime }}, & \mathrm{Target}\;\mathrm{is}\;\mathrm{in}\;\mathrm{cell}(r^{\prime },\theta^{\prime })\mathrm{of}\;\mathrm{the}\;k-\mathrm{th}\;\mathrm{frame},\\ w_{k}^{r,\theta }, & \mathrm{otherwise}, \end{array}\right. \end{array}\right.$$

where $r=1,\ldots ,N_{r},\;\theta =1,\ldots ,N_{\theta },\;k=1,\ldots ,K$,  $w_{k}^{r,\theta }$ is the clutter data, and

(11) $$A_{k}s_{k}=\frac{A_{k}}{\sqrt{N}}{\left[1,\exp\left(j2\pi f_{d_{k}}\right),\ldots ,\exp\left(j2\pi f_{d_{k}}\left(N-1\right)\right)\right]}^{T}$$

As observed in (10), it should be noted that the unknown target motion state poses significant challenges for detection. So, the kinematics boundary constraint (KBC) strategy is utilized to restrict the target state transition. This paper will be described in detail in Section 3.

### 2.3. Information Geometry Detector

Within the MIG detection framework, covariance matrices are frequently utilized to represent second-order statistical moments, thereby accurately reflecting the statistical properties of each sample data for the purpose of target detection. Specifically, the covariance matrix that captures the correlation features of the received signal $z={\left[z_{0},z_{1},\ldots ,z_{N-1}\right]}^{T}$ is modeled as the following Toeplitz structure,

(12) $$C=E\left[zz^{H}\right]=\left[\begin{array}{ccccccc} c_{0} & \; & {\bar{c}}_{1} & \; & \ldots & \; & {\bar{c}}_{N-1}\\ c_{1} & \; & c_{0} & \; & \ldots & \; & {\bar{c}}_{N-2}\\ \vdots & \; & \ddots & \; & \ddots & \; & \vdots \\ c_{N-1} & \; & c_{N-2} & \; & \ldots & \; & c_{0} \end{array}\right]$$

(13) $$c_{k}=E\left[z_{i}{\bar{z}}_{i+k}\right],\;0\leq k\leq N-1,\;0\leq i\leq N-k-1$$

where E(·) denotes the statistical expectation, ${\left(\cdot \right)}^{H}$ denotes conjugate transpose operators, and $c_{k}=E\left[z_{i}{\bar{z}}_{i+k}\right]$ indicates the correlation coefficient. Here, $c_{k}$ corresponds to the k-th correlation coefficient of the *z*, while ${\bar{c}}_{k}$ represents its conjugate. Leveraging the ergodicity property of stationary processes, the correlation coefficient $c_{k}$ can be estimated by

(14) $${\hat{c}}_{k}=\frac{1}{N}\sum_{j=0}^{N-k-1}z_{j}{\bar{z}}_{j+k},0\leq k\leq N-1$$

Following the aforementioned principles, each cell pulse signal is represented by a HPD matrix. This matrix can effectively represent the correlation and energy levels between different pulses.

Intuitively, manifolds are spaces that are locally, topologically equivalent to well-known Euclidean spaces. Consider the set P(n) of n×n Hermitian matrices, defined as $P\left(n\right)=\left\{C|C=C^{H}\right\}$. A matrix c is positive definite if $z^{H}Cz>0$ for all $z\in C^{n}\setminus \left\{0\right\}$. This property defines the subset $P^{+}\left(n\right)$ of HPD matrices:

(15) $$P^{+}\left(n\right)=\left\{C\in P\left(n\right)|C=C^{H},C≻0\right\}$$

This subset, $P^{+}\left(n\right)$, represents the space of HPD matrices. The geometric structure of this space is significant because it forms a matrix manifold, which is a concept from differential geometry where the space locally resembles Euclidean space. The matrix manifold $P^{+}\left(n\right)$ is particularly important in various fields such as optimization, signal processing, and control theory. It provides a framework for analyzing and applying HPD matrices in practical scenarios, leveraging their geometric properties to solve problems more effectively.

It should be highlighted that a metric elucidates the physical significance of points in a space being “near” or “distant” from one another. The geometric measure between any pair of points (matrices) on $P^{+}\left(n\right)$ is delineated. The geometric measure function (divergence) is $D:P^{+}\left(n\right)\times P^{+}\left(n\right)\to R$; it satisfies the following:

1. $D(C_{1},C_{2})\geq 0$, $\forall C_{1},C_{2}\in P^{+}\left(n\right)$.
2. $D(C_{1},C_{2})=0$ if and only if $C_{1}=C_{2}$.

It is important to note that for the geometric measure to qualify as a distance function, it must exhibit symmetry relative to the two matrices in question and adhere to the triangle inequality.

For the given set of HPD matrices $C_{1},C_{2},\ldots ,C_{N_{R}}$ on a matrix manifold, the geometric centroid is the solution to the following minimal problem:

(16) $$\begin{array}{cc} \hat{C} & =G(C_{1},C_{2},\ldots ,C_{N_{R}})\\ \; & =\arg\min_{C\in P^{+}\left(n\right)}\frac{1}{N_{R}}\sum_{k=1}^{N_{R}}\phi_{D}\left(C,C_{k}\right) \end{array}$$

**Figure 3:**
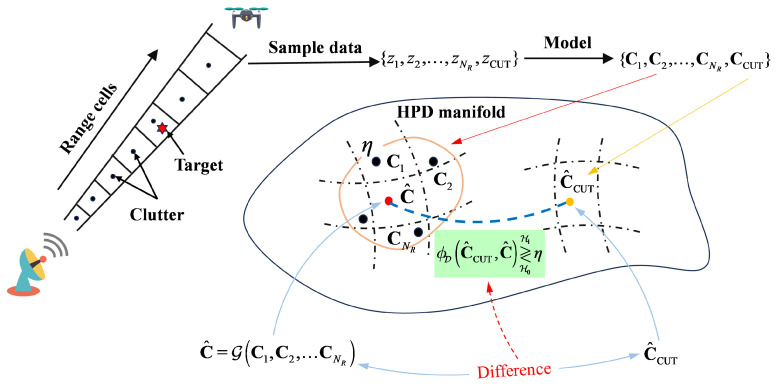
Framework of MIG detector in a coherent processing interval.

The MIG detector’s decision relies on a geometric measure between the covariance matrix ${\hat{C}}_{\mathrm{CUT}}$ of the CUT and the centroid $\hat{C}$ of matrices $\left(C_{1},C_{2},\ldots ,C_{N_{R}}\right)$ from reference cells expressed as

(17) $$\phi_{D}\left({\hat{C}}_{\mathrm{CUT}},\hat{C}\right)\underset{H_{0}}{\overset{H_{1}}{≷}}\eta$$

where $\phi_{D}$ is the geometric distance. The threshold η, typically derived through Monte Carlo simulations, is set according to the predetermined false alarm rate. Geometric distance serves as a metric to measure the difference between the target and clutter characteristics. In contrast to conventional energy-based detection approaches, the MIG detector’s determination relies on evaluating whether the geometric distance surpasses a predefined threshold, as referenced in [17]. The framework is illustrated in Figure 3.

The PSIG detector is constructed similarly to the MIG detector, except that the HPD manifold is replaced with a PS manifold. The derivation process is not repeated here, and details can be found in [23].

Similarly, the detection decision of the PSIG detector is

(18) $$\psi_{D}\left({\hat{P}}_{\mathrm{CUT}},\hat{P}\right)\underset{H_{0}}{\overset{H_{1}}{≷}}\eta^{\prime }$$

where $\eta^{\prime }$ is also the threshold and $\psi_{D}$ is the geometric distance.

According to reference [23], due to the universal applicability and performance advantages of the Kullback–Leibler (KL) divergence in matrix manifolds and power spectrum manifolds, KL divergence will be used as a metric on manifolds in the subsequent experiments of this paper. The KL divergence is a commonly used metric on manifolds which is a special case of information divergence. For two matrices $C_{1}$ and $C_{2}$, the KL divergence is defined as

(19) $$\phi_{D_{\mathrm{KL}}}\left(C_{1},C_{2}\right)=\mathrm{tr}\left(C_{2}^{-1}C_{1}-I\right)-\log\det\left(C_{2}^{-1}C_{1}\right)$$

where det(·) and tr(·) stand for the determinate and trace of a matrix. The KL divergence geometric centroid matrix is given as follows,

(20) $$\hat{C}={\left(\frac{1}{N_{R}}\sum_{k=1}^{N_{R}}C_{k}^{-1}\right)}^{-1}$$

It should be noted that the KL divergence does not satisfy the symmetry and triangular inequality.

## 3. IG-Based Track-Before-Detect Algorithm

In this section, kinematic constraints are first established for the trajectories of the moving targets. Then the scoring function is designed based on the IG framework. Finally, an IG-based DP-TBD algorithm for range-azimuth measurements is derived.

### 3.1. Kinematic Constraint for Trajectory of Moving Target

**Figure 4:**
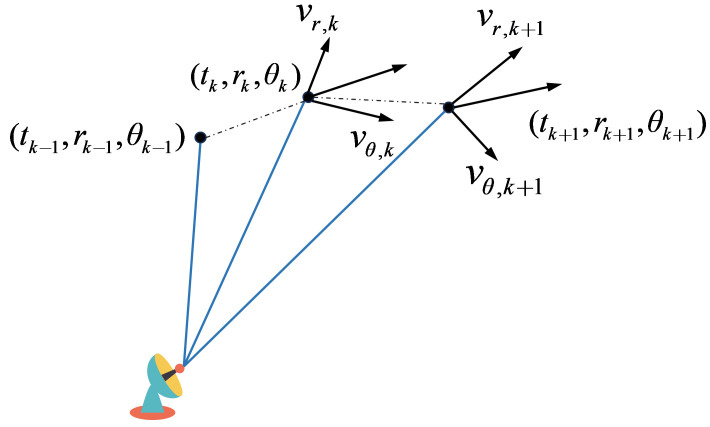
Inter-frame target position versus velocity.

Each target must adhere to certain kinematic constraints, which restrict the extent of the range for inter-frame state transitions. The constraints considered here are the maximum target velocity $v_{max}$ and the maximum target acceleration $a_{max}$. Consider two adjacent data frames, with the target state shown in Figure 4. Hence, considering the kinematic constraints,

(21) $$\begin{array}{cc} \; & {\left|{\dot{r}}_{k+1}\right|}^{2}+{\left|{\dot{\theta }}_{k+1}\right|}^{2}<v_{max}^{2} \end{array}$$

(22) $$\begin{array}{cc} \; & {\left|{\ddot{r}}_{k+1}\right|}^{2}+{\left|{\ddot{\theta }}_{k+1}\right|}^{2}<a_{max}^{2} \end{array}$$

where ${\dot{r}}_{k+1}$ and ${\dot{\theta }}_{k+1}$ are instantaneous velocities, and ${\ddot{r}}_{k+1}$ and ${\ddot{\theta }}_{k+1}$ are instantaneous accelerations, in the *r* and θ dimensions, respectively.

Based on measurements at the k-th frame $\left(t_{k},r_{k},\theta_{k}\right)$ and k+1-th frame $\left(t_{k+1},r_{k+1},\theta_{k+1}\right)$, it can be easily shown that the radial velocities ${\dot{r}}_{k+1}$ and tangential velocities ${\dot{\theta }}_{k+1}$ at the k+1-th frame can be estimated by $v_{r,k+1}$ in (23) and $v_{\theta ,k+1}$ in (24), respectively.

(23) $$\begin{array}{cc} v_{r,k+1} & =\frac{r_{k+1}-r_{k}\cos\left(\theta_{k+1}-\theta_{k}\right)}{t_{k+1}-t_{k}} \end{array}$$

(24) $$\begin{array}{cc} v_{\theta ,k+1} & =\frac{r_{k}\sin\left(\theta_{k+1}-\theta_{k}\right)}{t_{k+1}-t_{k}} \end{array}$$

where $t_{k}$ and $t_{k+1}$ represent the moments when the k-th frame and the (k+1)-th frame are captured, and $t_{k+1}-t_{k}$ is the scanning period *T*.

However, since the range and azimuth measurements are prone to errors, these inaccuracies will inevitably propagate to the velocity estimates. Assuming that the standard deviations of *r* and θ errors are $\sigma_{r}$ and $\sigma_{\theta }$, and these errors have zero mean, the standard deviation of the velocity errors in (23) and (24) are approximated as

(25) $$\begin{array}{cc} \sigma_{v_{r,k+1}} & =\frac{\sqrt{\left(1+\cos^{2}\left(\theta_{k+1}-\theta_{k}\right)\right)\sigma_{r}^{2}+2r_{k}^{2}\sin^{2}\left(\theta_{k+1}-\theta_{k}\right)\sigma_{\theta }^{2}}}{t_{k+1}-t_{k}} \end{array}$$

(26) $$\begin{array}{cc} \sigma_{v_{\theta ,k+1}} & =\frac{\sqrt{\sin^{2}\left(\theta_{k+1}-\theta_{k}\right)\sigma_{r}^{2}+2r_{k}^{2}\cos^{2}\left(\theta_{k+1}-\theta_{k}\right)\sigma_{\theta }^{2}}}{t_{k+1}-t_{k}} \end{array}$$

Hence, the velocity constraint (21) becomes

(27) $${\left[{\left(\left|v_{r,k+1}\right|-\beta \sigma_{v_{r,k+1}}\right)}^{+}\right]}^{2}+{\left[{\left(\left|v_{\theta ,k+1}\right|-\beta \sigma_{v_{\theta ,k+1}}\right)}^{+}\right]}^{2}<v_{\max}^{2}$$

where $x^{+}=max\left\{x,0\right\}$, and β accounts for a given percentage of the errors. In the case of a Gaussian distribution, approximately 95.5% and 99.7% of errors fall within β=2 and β=3 standard deviations of the mean, respectively. When the error distribution is uncertain, Chebyshev’s inequality provides a useful alternative for estimation purposes [41].

For acceleration constraints, three consecutive frames of measurements are used. $v_{r,k}$ and $v_{\theta ,k}$ can be obtained using the measurements at the k−1-th frame $\left(t_{k-1},r_{k-1},\theta_{k-1}\right)$ and k-th frame $\left(t_{k},r_{k},\theta_{k}\right)$. It is clear that the radial accelerations ${\ddot{r}}_{k+1}$ and tangential accelerations ${\ddot{\theta }}_{k+1}$ at the k+1-th frame $\left(t_{k+1},r_{k+1},\theta_{k+1}\right)$ can be estimated by $a_{r,k+1}$ in (28) and $a_{\theta ,k+1}$ in (29), respectively.

(28) $$\begin{array}{cc} a_{r,k+1} & =\frac{v_{r,k+1}-\left(v_{r,k}\cos\left(\theta_{k+1}-\theta_{k}\right)+v_{\theta ,k}\sin\left(\theta_{k+1}-\theta_{k}\right)\right)}{t_{k+1}-t_{k}}\\ \; & =\frac{r_{k+1}-r_{k}\cos\left(\theta_{k+1}-\theta_{k}\right)}{{\left(t_{k+1}-t_{k}\right)}^{2}}-\frac{r_{k}\cos\left(\theta_{k+1}-\theta_{k}\right)-r_{k-1}\cos\left(\theta_{k+1}-\theta_{k-1}\right)}{\left(t_{k+1}-t_{k}\right)\left(t_{k}-t_{k-1}\right)} \end{array}$$

(29) $$\begin{array}{cc} a_{\theta ,k+1} & =\frac{v_{\theta ,k+1}-\left(-v_{r,k}\sin\left(\theta_{k+1}-\theta_{k}\right)+v_{\theta ,k}\cos\left(\theta_{k+1}-\theta_{k}\right)\right)}{t_{k+1}-t_{k}}\\ \; & =\frac{r_{k}\sin\left(\theta_{k+1}-\theta_{k}\right)}{{\left(t_{k+1}-t_{k}\right)}^{2}}-\frac{-r_{k}\sin\left(\theta_{k+1}-\theta_{k}\right)+r_{k-1}\sin\left(\theta_{k+1}-\theta_{k-1}\right)}{\left(t_{k+1}-t_{k}\right)\left(t_{k}-t_{k-1}\right)} \end{array}$$

Considering the errors in radial and tangential acceleration, the approximate expression for the standard deviations are

(30) $$\begin{array}{c} \sigma_{a_{r,k+1}}=\frac{1}{t_{k+1}-t_{k}}\left\{\left[\frac{1}{{\left(t_{k+1}-t_{k}\right)}^{2}}+\cos^{2}\left(\theta_{k+1}-\theta_{k}\right){\left(\frac{1}{t_{k+1}-t_{k}}+\frac{1}{t_{k}-t_{k-1}}\right)}^{2}+\frac{\cos^{2}\left(\theta_{k+1}-\theta_{k-1}\right)}{{\left(t_{k}-t_{k-1}\right)}^{2}}\right]\sigma_{r}^{2}\right.\\ +\left.\left[{\left(r_{k}\sin\left(\theta_{k+1}-\theta_{k}\right)\left(\frac{1}{t_{k+1}-t_{k}}+\frac{1}{t_{k}-t_{k-1}}\right)-\frac{r_{k-1}\sin\left(\theta_{k+1}-\theta_{k-1}\right)}{t_{k}-t_{k-1}}\right)}^{2}\right.\right.\\ +{\left.\left.r_{k}^{2}\sin^{2}\left(\theta_{k+1}-\theta_{k}\right){\left(\frac{1}{t_{k+1}-t_{k}}+\frac{1}{t_{k}-t_{k-1}}\right)}^{2}+\frac{r_{k-1}^{2}\sin^{2}\left(\theta_{k+1}-\theta_{k-1}\right)}{{\left(t_{k}-t_{k-1}\right)}^{2}}\right]\sigma_{\theta }^{2}\right\}}^{\frac{1}{2}} \end{array}$$

(31) $$\begin{array}{c} \sigma_{a_{\theta ,k+1}}=\frac{1}{t_{k+1}-t_{k}}\left\{\left[\sin^{2}\left(\theta_{k+1}-\theta_{k}\right){\left(\frac{1}{t_{k+1}-t_{k}}+\frac{1}{t_{k}-t_{k-1}}\right)}^{2}\right.\right.\left.+\frac{\sin^{2}\left(\theta_{k+1}-\theta_{k-1}\right)}{{\left(t_{k}-t_{k-1}\right)}^{2}}\right]\sigma_{r}^{2}\\ +\left[\left(r_{k}\cos\left(\theta_{k+1}-\theta_{k}\right)\left(\frac{1}{t_{k+1}-t_{k}}+\frac{1}{t_{k}-t_{k-1}}\right)\right.\right.{\left.-\frac{r_{k-1}\cos\left(\theta_{k+1}-\theta_{k-1}\right)}{t_{k}-t_{k-1}}\right)}^{2}\\ +r_{k}^{2}\cos^{2}\left(\theta_{k+1}-\theta_{k}\right){\left(\frac{1}{t_{k+1}-t_{k}}+\frac{1}{t_{k}-t_{k-1}}\right)}^{2}+{\left.\left.\frac{r_{k-1}^{2}\cos^{2}\left(\theta_{k+1}-\theta_{k-1}\right)}{{\left(t_{k}-t_{k-1}\right)}^{2}}\right]\sigma_{\theta }^{2}\right\}}^{\frac{1}{2}} \end{array}$$

So, the acceleration constraint (22) becomes

(32) $${\left[{\left(\left|a_{r,k+1}\right|-\beta \sigma_{a_{r,k+1}}\right)}^{+}\right]}^{2}+{\left[{\left(\left|a_{\theta ,k+1}\right|-\beta \sigma_{a_{\theta ,k+1}}\right)}^{+}\right]}^{2}<a_{max}^{2}$$

By the aforementioned derivation, the range of target state transitions can be solved frame by frame, utilizing the constraints on the target’s maximum velocity and maximum acceleration in (27) and (32).

After the velocity and acceleration constraints, the state transfer relationship between adjacent frames can be solved. We use Λ to denote the discrete state search region. With a source state ${\hat{x}}_{k-1}$ at frame k−1, a potential destination state region at frame *k* is defined as

(33) $$\begin{array}{c} \Lambda \left({\hat{x}}_{k-1}\right)=\left\{{\hat{x}}_{k}\in R^{4}:{\left|{\dot{r}}_{k}\right|}^{2}+{\left|{\dot{\theta }}_{k}\right|}^{2}<v_{max}^{2}\;\mathrm{and}\;{\left|{\ddot{r}}_{k}\right|}^{2}+{\left|{\ddot{\theta }}_{k}\right|}^{2}<a_{max}^{2}\right\} \end{array}$$

Similarly, for each destination state ${\hat{x}}_{k}$ at frame k, a region of potential source states is defined to identify which source state at frame k−1 could transition to ${\hat{x}}_{k}$

(34) $$\Lambda^{-1}\left({\hat{x}}_{k}\right)=\left\{{\hat{x}}_{k-1}\in R^{4}:{\hat{x}}_{k}\in \Lambda \left({\hat{x}}_{k-1}\right)\right\}$$

In particular, $\Lambda \left({\hat{x}}_{k-1}\right)$ offers a precise grid state search area in the range-azimuth measurements, which effectively accounts for the effects of noise and measurement errors in real processes. With this gridded approach, the state transition region can be predicted more accurately, improving overall accuracy and reliability.

### 3.2. IG-Based Scoring Function

**Figure 5:**
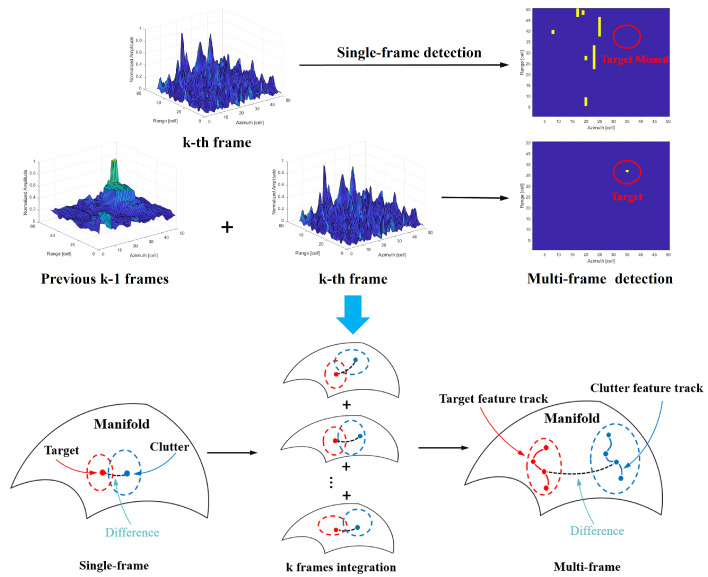
Transition from single-frame detection to multi-frame detection.

Single-frame detection only utilizes the information from the current frame for detection. When the clutter is strong, the target will be missed. In contrast, multi-frame detection leverages the integrated information from the previous k−1 frames. After the target information is integrated over *k* frames, the target becomes easier to detect. On the manifold, the integration of differences between multiple frames manifests as the integration of distances between the target and clutter, which is shown in Figure 5.

**Figure 6:**
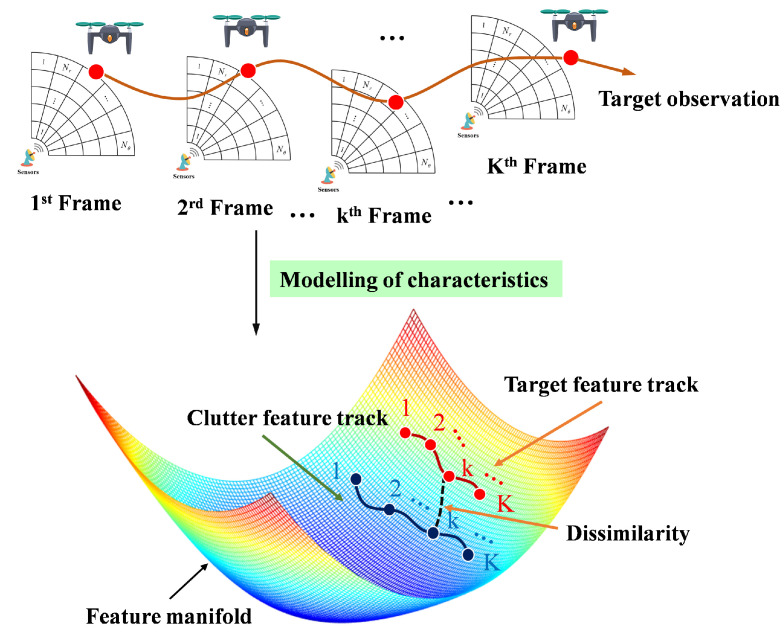
Multi-frame feature dissimilarity of targets and clutter on the manifolds.

Based on the principle of the IG detector, the radar echo signals are mapped onto the manifold. The observation data of the *k*-th frame is modeled as a covariance matrix or a power spectrum. Along with the target motion track, the target and clutter features also form two tracks on the manifolds as depicted in Figure 6. The track of the target on the manifold represents the similarity of the target’s features. The distance between the clutter track and the target track on the manifold represents the difference in features between the clutter and the target. Denote the target state estimate by ${\hat{X}}_{1:K}=\left\{{\hat{x}}_{1},\ldots ,{\hat{x}}_{K}\right\}$, where ${\hat{x}}_{k}=\left(r_{k},\theta_{k}\right)$ denotes the resolution cell containing a target in each frame.

Since the process is similar for MIG and PSIG in the subsequent derivation, R and D will be used in the formulas in this section instead of C, $\phi_{D}$ in (17) and P, $\psi_{D}$ in (18).

In the IG detection framework, in order to fully exploit the feature dissimilarity of targets and clutter on the manifolds, the target state estimate should satisfy the following two properties:

(1) For each frame 1≤k≤K, the distance between the target cells and clutter cells of the intra-frame $D\left({\hat{R}}_{\mathrm{CUT}}^{k}\left(r_{k},\theta_{k}\right),{\hat{R}}^{k}\left(r_{k},\theta_{k}\right)\right)$ should be as far as possible on the manifold, which is the dissimilarity in the Figure 6, i.e.,
  (35) $$\begin{array}{cc} \arg\max_{\overset{\left({\hat{x}}_{1},\ldots ,{\hat{x}}_{K}\right)}{{\hat{x}}_{k}\in \Lambda \left({\hat{x}}_{k-1}\right)}} & \sum_{k=1}^{K}D\left({\hat{R}}_{\mathrm{CUT}}^{k}\left(r_{k},\theta_{k}\right),{\hat{R}}^{k}\left(r_{k},\theta_{k}\right)\right) \end{array}$$
(2) For adjacent frames 1≤k−1,k≤K, the distance between the target cells in adjacent frames $D\left({\hat{R}}_{\mathrm{CUT}}^{k}\left(r_{k},\theta_{k}\right),{\hat{R}}_{\mathrm{CUT}}^{k-1}(r_{k-1},\theta_{k-1}\right)$ needs to keep as close as possible on the manifold, which is the target feature track in the Figure 6, i.e.,
  (36) $$\begin{array}{cc} \arg\min_{\overset{\left({\hat{x}}_{1},\ldots ,{\hat{x}}_{K}\right)}{{\hat{x}}_{k}\in \Lambda \left({\hat{x}}_{k-1}\right)}} & \sum_{k=2}^{K}D\left({\hat{R}}_{\mathrm{CUT}}^{k}\left(r_{k},\theta_{k}\right),{\hat{R}}_{\mathrm{CUT}}^{k-1}(r_{k-1},\theta_{k-1}\right) \end{array}$$
  where $\Lambda ({\hat{x}}_{k-1})$ is a possible destination state region in the *k*-th frame in (33).

The weighted sum method is a multi-objective optimization technique that simplifies the problem by combining multiple objective functions into a single objective function. By adjusting these weights, it is possible to control the trade-offs between the different objectives in the optimization process, thus achieving a balance between multiple objectives. So, combining these two optimization problems, the proposed multi-objective optimization framework is rewritten as

(37) $$\begin{array}{c} {\hat{X}}_{1:K}=\arg\max_{\overset{\left({\hat{x}}_{1},\ldots ,{\hat{x}}_{K}\right)}{{\hat{x}}_{k}\in \Lambda \left({\hat{x}}_{k-1}\right)}}\left(\left(1-\alpha \right)\sum_{k=1}^{K}D\left({\hat{R}}_{\mathrm{CUT}}^{k}\left(r_{k},\theta_{k}\right),{\hat{R}}^{k}\left(r_{k},\theta_{k}\right)\right)-\alpha \sum_{k=2}^{K}D\left({\hat{R}}_{\mathrm{CUT}}^{k}\left(r_{k},\theta_{k}\right),{\hat{R}}_{\mathrm{CUT}}^{k-1}(r_{k-1},\theta_{k-1}\right)\right) \end{array}$$

Therefore, the scoring function based on the information geometry for ${\hat{x}}_{k}\in R^{4}$, 2≤k≤K is defined as

(38) $$\begin{array}{c} I\left({\hat{x}}_{k}\right)=\left(1-\alpha \right)D\left({\hat{R}}_{\mathrm{CUT}}^{k}\left(r_{k},\theta_{k}\right),{\hat{R}}^{k}\left(r_{k},\theta_{k}\right)\right)-\alpha D\left({\hat{R}}_{\mathrm{CUT}}^{k}\left(r_{k},\theta_{k}\right),{\hat{R}}_{\mathrm{CUT}}^{k-1}\left(r_{k-1},\theta_{k-1}\right)\right) \end{array}$$

For the boundary case of k=1, the algorithm operationally collapses into a single-frame detection framework, thereby aligning with conventional frame-wise processing paradigms.

Therefore, using the proposed IG-based scoring function, multi-frame detection and target trajectory estimation can be implemented in the next subsection.

### 3.3. Multi-Frame Detection and Trajectory Estimate

Given the first-order Markovian property governing target state transitions, the integrated merit function (IMF) $J({\hat{x}}_{k})$ over 2≤k≤K is recursively constructed as the extremum of a nested optimization subproblem,

(39) $$J\left({\hat{x}}_{k}\right)=\max_{\overset{\left({\hat{x}}_{1},\ldots ,{\hat{x}}_{K}\right)}{{\hat{x}}_{k}\in \Lambda \left({\hat{x}}_{k-1}\right)}}\sum_{i=2}^{k}I\left({\hat{x}}_{i}\right)$$

Thus, the IMF $J({\hat{x}}_{k})$ signifies the maximal merit value across all trajectories $\left({\hat{x}}_{1},\ldots ,{\hat{x}}_{K}\right)$ that adhere to the condition ${\hat{x}}_{k}\in \Lambda \left({\hat{x}}_{k-1}\right)$.

The IMF can be computed recursively using a DP algorithm by exploiting the first-order Markov property. For 2≤k≤K, the IMF $J({\hat{x}}_{k})$ of ${\hat{x}}_{k}\in R^{4}$ is computed sequentially using DP algorithm:

(40) $$\begin{array}{c} J\left({\hat{x}}_{k}\right)=\max_{{\hat{x}}_{k-1}\in \Lambda^{-1}\left({\hat{x}}_{k}\right)}J\left({\hat{x}}_{k-1}\right)+I\left({\hat{x}}_{k}\right) \end{array}$$

The DP algorithm obtains the complete IMF $J({\hat{x}}_{k})$ recursively by leveraging the first-order Markovian property, and the state transition equation is

(41) $$\begin{array}{c} J\left({\hat{x}}_{k}\right)=\left\{\begin{array}{cc} \max_{{\hat{x}}_{k-1}\in \Lambda^{-1}\left({\hat{x}}_{k}\right)}J\left({\hat{x}}_{k-1}\right)+I\left({\hat{x}}_{k}\right), & k>1,\\ D\left({\hat{R}}_{\mathrm{CUT}}^{k}\left(r_{k},\theta_{k}\right),{\hat{R}}^{k}\left(r_{k},\theta_{k}\right)\right), & k=1. \end{array}\right. \end{array}$$

**Figure 7:**
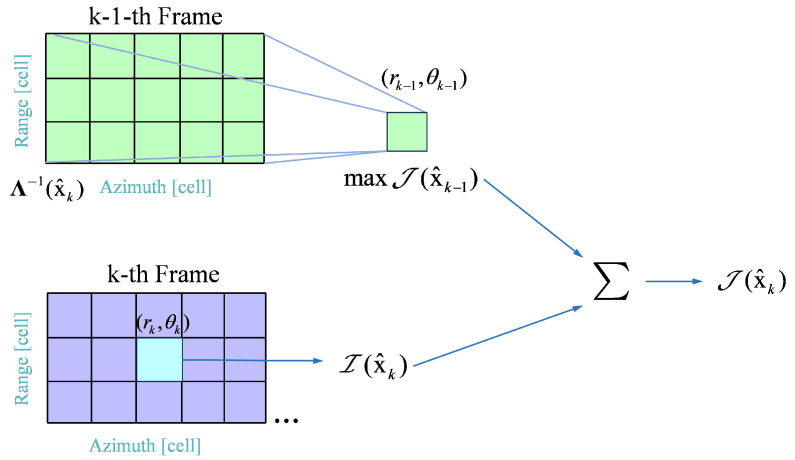
The state transition of dynamic programming.

Equation (41) represents a recursive integration process, focusing solely on the grid transition relationship between adjacent frames. This process of state transfer of the DP algorithm is shown in Figure 7. Multi-frame integrated based on kinematic constraints in the search region $\Lambda^{-1}\left({\hat{x}}_{k}\right)$ suppresses a large number of spurious paths. This makes it possible to perform long-time integration in the range-azimuth domain.

The target detection for multi-frame detection can be expressed as

(42) $$\max_{{\hat{x}}_{k}\in R^{4}}J\left({\hat{x}}_{K}\right)\overset{H_{1}}{\underset{H_{0}}{≷}}\eta_{K}$$

where $H_{1}$ denotes the hypothesis under which target exists and which target trajectory ${\hat{X}}_{1:K}=\left\{{\hat{x}}_{1},\ldots ,{\hat{x}}_{K}\right\}$ is generated. $H_{0}$ indicates that the target does not exist in the observation area. The detection threshold $\eta_{K}$ is set under the Neyman–Pearson criterion to satisfy a predefined false alarm probability $P_{fa}$.

The target trajectory for 2≤k≤K can be estimated recursively by the following recursive formula, where $W({\hat{x}}_{k})$ is the recursive formula for the target trajectory,

(43) $$\begin{array}{c} W\left({\hat{x}}_{k}\right)=\arg\max_{{\hat{x}}_{k-1}\in \Lambda^{-1}\left({\hat{x}}_{k}\right)}J\left({\hat{x}}_{k-1}\right),{\hat{x}}_{k}\in R^{4} \end{array}$$

Specifically, the target trajectory is estimated recursively using the following equation:

(44) $$\begin{array}{c} {\hat{x}}_{k}=\left\{\begin{array}{cc} W({\hat{x}}_{k+1}), & k=1,\ldots ,K-1,\\ \arg\max_{\overset{1\leq \hat{r}\leq N_{r}}{1\leq \hat{\theta }\leq N_{\theta }}}J\left({\hat{x}}_{k}\right), & k=K. \end{array}\right. \end{array}$$

Algorithm 1 also gives the pseudocode of the IG-based DP-TBD algorithm for range-azimuth Measurements.

**Algorithm 1:** IG-based DP-TBD algorithm for range-azimuth Measurements

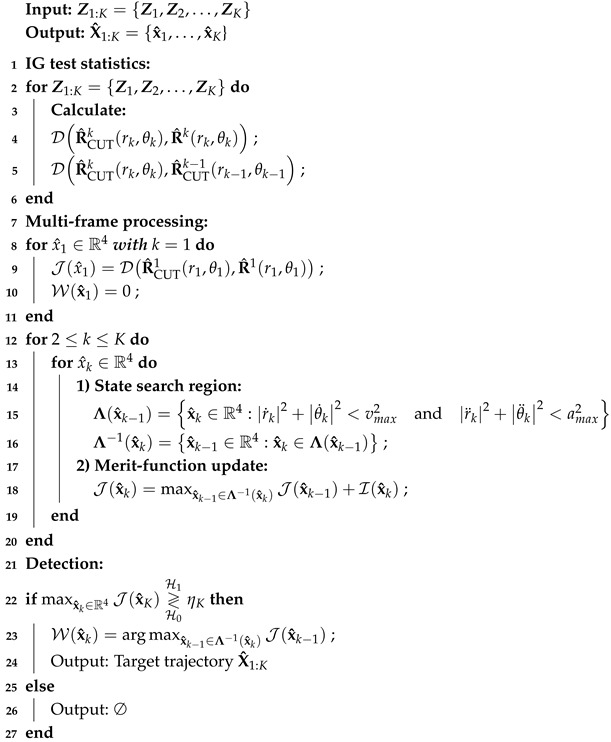



### 3.4. Computation Complexity

For Algorithm 1, the computational complexity of the DP-TBD algorithm based on information geometry mainly comes from the scoring function and merit function calculations.

For the calculation of the scoring function, the computational expense of the MIG detector based on the KL divergence mainly depends on the calculation of the geometric centroid (20), hence its computational complexity is $O(N_{R}N^{3})$. The computational cost of the PSIG detector based on the KL divergence mainly depends on the calculation of the power spectrum, and its computational complexity is $O(N_{R}N\log N)$. Additionally, the number of elements in the search region obtained by the kinematic constraint method is denoted as |Λ|. Therefore, the number of merit function calculations is $KN_{r}N_{\theta }\left|\Lambda \right|$. In summary, the computational complexity of the MIG-based DP-TBD algorithm is $O(KN_{r}N_{\theta }|\Lambda |N_{R}N^{3})$, and the computational complexity of the PSIG-based DP-TBD algorithm is $O(KN_{r}N_{\theta }|\Lambda |N_{R}N\log N)$.

## 4. Experimental Results and Analysis

In this section, real-recorded data are used to verify the validity of the IMF based on information geometry. Subsequently, the accuracy of the target trajectories estimate and detection performance advantages are verified. Evaluation metrics are conducted based on the following three measures:

- **Target detection probability $P_{d}$:** the maximum value of the IMF exceeds the detection threshold, while the discrepancy between the estimated and actual target position in the final frame is under two cells.
- **Target track detection probability $P_{d-\mathrm{track}}$**: the maximum value of the IMF exceeds the threshold, and the error of the estimated target trajectory compared with the real target trajectory is less than two cells within the whole track duration.
- **Root Mean Square Error of Position RMSE**: the average position error between the estimated trajectory and the real trajectory in the Cartesian Coordinate System.
  (45) $$RMSE=\sqrt{\frac{\sum_{n=1}^{N}\sum_{k=1}^{K}\left({\left({\hat{r}}_{k}-r_{k}\right)}^{2}+{\left({\hat{\theta }}_{k}-\theta_{k}\right)}^{2}\right)}{NK}}$$
  *N* denotes the number of Monte Carlo simulations.

For comparison purposes, the performance of the proposed MIG-DP-TBD and PSIG-DP-TBD methods are contrasted with several traditional methods. These methods include the traditional FFT-based coherent integration method, the squared amplitude (SA) based incoherent integration method [42], the popular adaptive matched filter (AMF) method, and the novel maximal eigenvalue (ME) method [33], and are considered for multi-frame TBD, which are marked as FFT-DP-TBD, SA-DP-TBD, AMF-DP-TBD, and ME-DP-TBD, respectively.

### 4.1. Real-Recorded Sea Clutter Data

**Figure 8:**
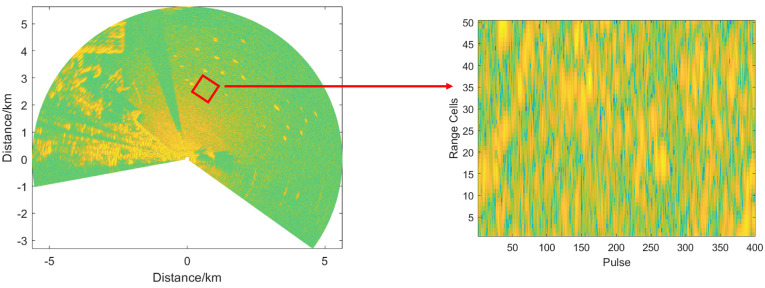
Measured data and intercepted frame of data.

This section utilizes the data from the ‘Dataset of Radar Detecting Sea’ [43,44], which were collected in 2019 and 2021. The experimental data was obtained at Yangma Island, Yantai, under sea state 3–4. The radar worked in scanning mode. The specific radar parameters are shown in Table 1. Only linear frequency modulation data are considered in this section. A portion of the observation area is selected to form a frame as shown in Figure 8.

The CPI consists of N=8 pulses, with $N_{R}=8$ reference cells utilized. The parameter α in the dynamic programming is set to be α=0.01. In order to verify the multi-frame detection performance and trajectory estimation accuracy, a simulation target in (11) is added to the sea clutter data to obtain its trajectory information. In addition, the amplitude $A_{k}$ of the target echo at different frames will be set as a zero-mean Gaussian random variable whose variance is associated with the SCR. The target motion model is set according to (5). Its initial velocity is a random variable following a uniform distribution within the range of [−30, 30] m/s. The upper bound of the target motion velocity is set to 30 m/s. The noisy “acceleration” $v_{r}$ and $v_{\theta }$ in (7) follows a uniform distribution across the interval from −2.5 to 2.5 $\left(m/s^{2}\right)$.

### 4.2. Comparison of Integration Merit Function and Trajectory Estimate

**Figure 9:**
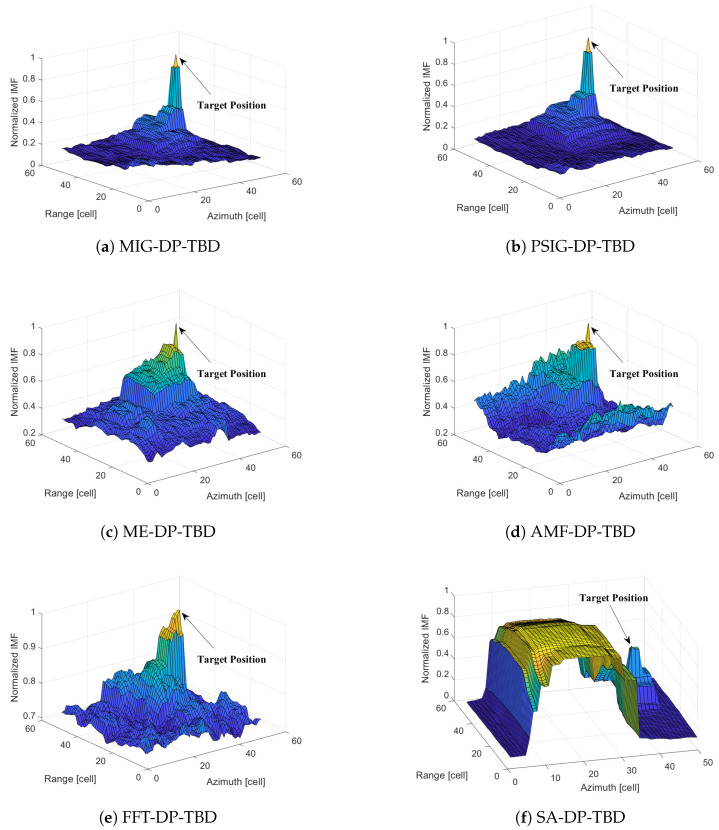
The normalized IMFs of the proposed methods and the comparison methods after integrating 15 frames.

Six different methods (MIG, PSIG, ME, AMF, FFT, and SA) are used to integrate the 15 frames data. Specifically, the used data files are 20191012110708_01_*scanning.mat* ∼ 20191012110735_15_*scanning.mat*. The pulse repetition frequency is 3000 Hz. 4440∼4839 pulses and 4001∼4050 range cells are selected to form a frame in this experiment. The SCR of the simulation target was set to 13 dB, resulting in the normalized integrated merit functions shown in Figure 9.

Through comparative analysis, we observe that the IG-based integration methods (MIG-based and PSIG-based) perform better in suppressing clutter. They effectively integrate the merit functions at the target position. When the merit functions reach their maximum values, the target is accurately identified. Consequently, the specific location information of the target is obtained. The DP algorithm allows backtracking to the resolution cell where the target is located in each frame, thus outputting the target’s motion trajectory during the observation time. In contrast, the comparison methods (including ME-based, AMF-based, FFT-based, and SA-based) do not perform well in clutter suppression because they also integrate the energy of strong clutter, which can lead to false alarms. The ME-based, AMF-based, and FFT-based methods are highly susceptible to strong clutter. Their integrated peak envelopes face serious energy expansion problems. These issues easily lead to target tracking errors. The traditional SA-based IMF directly integrates the echo energy and is the least effective. Strong clutter can directly mask the target.

**Figure 10:**
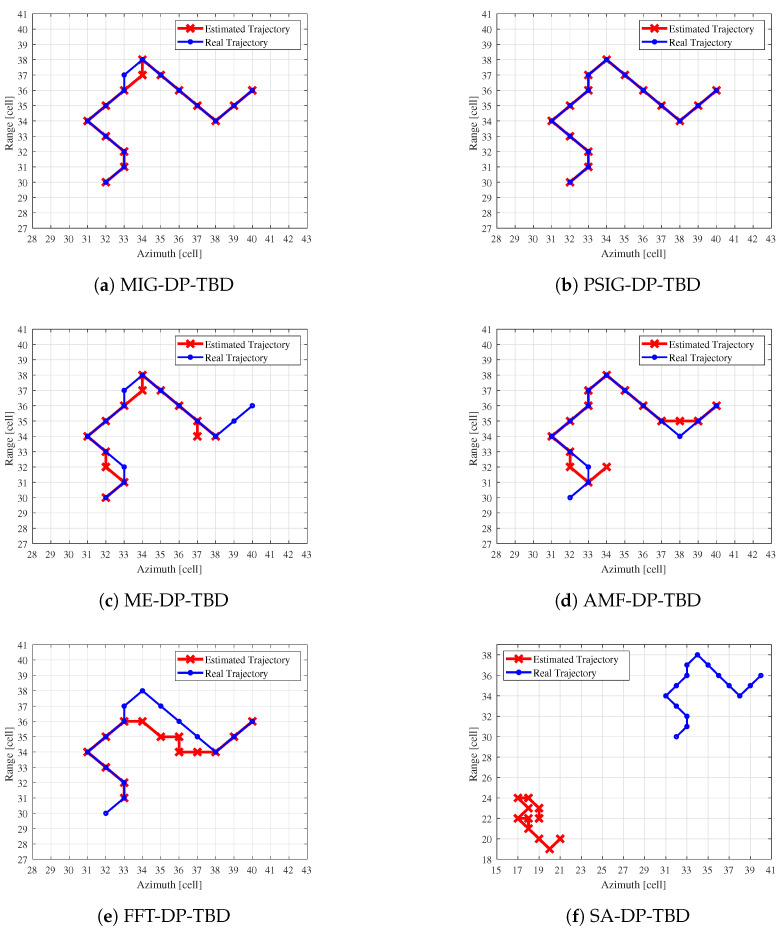
The estimated trajectories obtained by backtracking from the normalized IMFs after integrating 15 frames, as well as a comparison with real target trajectories.

Six different methods (MIG, PSIG, ME, AMF, FFT, and SA) are used to integrate the 15 frames data, resulting in the estimated trajectories obtained by backtracking from the maximum point of the normalized integrated merit functions shown in Figure 10.

Setting the SCR at 13 dB facilitates a more effective comparison of the estimated trajectories. The IG-based DP-TBD methods can effectively distinguish the clutter from the target to achieve effective integration of target information. The estimated trajectory derived from the IG method aligns closely with the real trajectory of the target, thereby demonstrating superior tracking performance. The corresponding indicators for the comparison methods is unsatisfactory, often resulting in the output of false point traces. The target trajectory points generated by the ME, AMF, and FFT methods all exhibit varying degrees of false point trajectories, with partial discrepancies observed between these trajectories and the actual target path. Moreover, the estimated target track points from the traditional SA-based method form a completely false track, failing to detect the target altogether.

### 4.3. Comparison of Target Detection and Track Performance

To verify the detection and tracking capabilities for moving targets amidst sea clutter, an experimental scenario is constructed, utilizing 10 frames of real-recorded sea clutter data. Specifically, the used data files are 20210106163953_01_*scanning.mat* ∼ 20210106164255_01_*scanning.mat*. The pulse repetition frequency is 1525 Hz. 1440∼2239 pulses and 1001∼1050 range cells are selected to form a frame in this experiment. Set the target to follow the CA model motion in the observation area with unknown and constant acceleration and $P_{fa}=10^{-3}$. The remaining parameters are the same as in Section 4.1.

**Figure 11:**
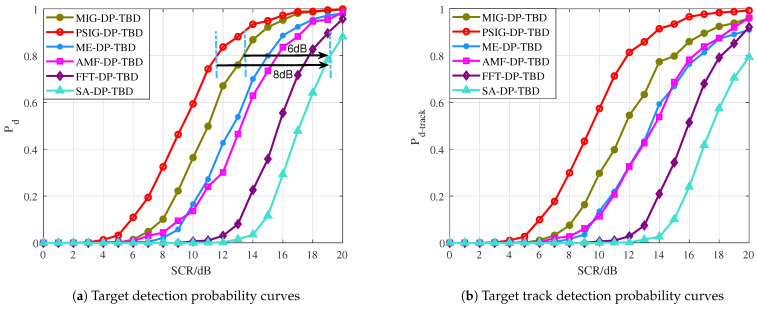
The target detection probability and track detection probability curves of the proposed methods versus the comparison methods.

In Figure 11a, the curves of $P_{d}$ versus target SCR is illustrated. As expected, the IG-based DP-TBD methods show robust detection performance with 6 dB to 8 dB SCR gain compared to SA-DP-TBD due to the novel IMF of the target between multiple consecutive frames. Moreover, the proposed PSIG-DP-TBD method shows superior performance, which improves about 3 dB over the ME-DP-TBD, about 3.5 dB over the AMF-DP-TBD, about 6 dB over the FFT-DP-TBD, and about 7 dB over the conventional SA-DP-TBD, when $P_{d}=0.8$. Compared with the well-performed ME-DP-TBD and AMF-DP-TBD, the proposed method MIG-DP-TBD improves about 2 dB when $P_{d}=0.8$. In addition, it improves about 4.5 dB and 5.5 dB over the FFT-DP-TBD and SA-DP-TBD, respectively. Hence, the PSIG-DP-TBD and MIG-DP-TBD perform better than the comparison methods. The IG-based DP-TBD algorithm improves the SCR by about 3∼7 dB compared to the general TBD algorithm.

The target tracking performance is given in Figure 11b. Figure 11b reports the curves of target track detection probability $P_{d-track}$ against the target SCR. The overall trend indicates that the $P_{d-track}$ increases with the SCR. Among the algorithms compared, the PSIG-DP-TBD exhibits the best tracking performance, followed by the MIG-DP-TBD. The ME-DP-TBD and the AMF-DP-TBD rank subsequently in terms of tracking effectiveness. It can be seen that the performance gap between the IG-based DP-TBD and the comparison methods in terms of effective detection of the entire target trajectory is consistent with the difference in $P_{d}$ in Figure 11a. This is understandable because as the target is detected in the last frame, the performance of its trajectory estimation will also improve.

**Figure 12:**
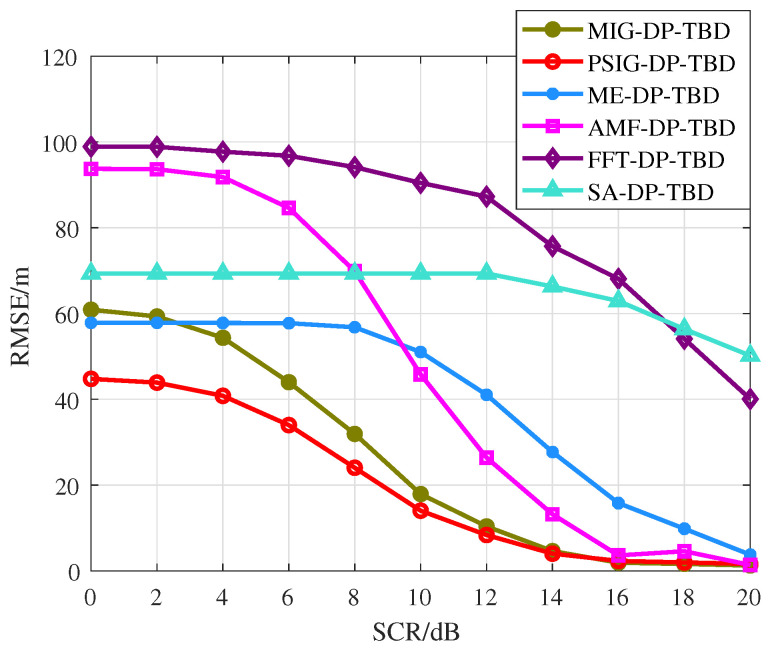
Root mean square errors of position for target trajectory estimation.

Figure 12 reports the RMSE versus the target SCR. The RMSE of all the methods decrease as the SCR increases, indicating the improvement in the accuracy of the trajectory estimation. Meanwhile, it is noteworthy that the RMSE of the PSIG-DP-TBD method is consistently lower, indicating both the accuracy of target trajectory estimation and the robustness of detection. The second best is the MIG-DP-TBD method, which demonstrates that the proposed method effectively integrates multi-frame information. It is observed that under low SCR conditions, certain comparison methods, such as SA-DP-TBD, maintain a constant RMSE. This phenomenon occurs because the target remains undetected, causing the algorithm to continuously output the same erroneous trajectory. The above results highlight the superiority of the proposed methods in detecting and tracking targets in a strong clutter background.

**Figure 13:**
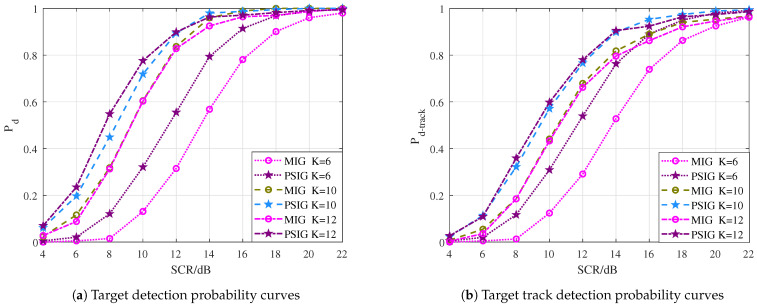
The target detection probability and track detection probability curves of PSIG-DP-TBD and MIG-DP-TBD with different K.

In order to analyze the improvement of detection performance by inter-frame integration, the $P_{d}$ and $P_{d-track}$ curves of the proposed methods are given in Figure 13 with different numbers of frames. Specifically, the used data files are 20191012110708_09_*scanning.mat* ∼ 20191012110757_20_*scanning.mat*. The pulse repetition frequency is 3000 Hz. 3440∼3839 pulses and 4001∼4050 range cells are selected to form a frame in this experiment. The number of consecutive frames is set to K = 6, K = 10, and K = 12, respectively.

As shown in Figure 13, it can be noted that both methods achieve improved detection performance as K increases, which indicates that inter-frame signal integration has been achieved and demonstrates the effectiveness of the multi-frame detection method. However, computational cost increases dramatically as K increases. Meanwhile, the improvement in performance resulting from an increase in the number of frames is also limited. As the number of frames increases, the uncertainty in the target state gradually converges. Consequently, the information provided by additional frames becomes increasingly redundant. Meanwhile, the ability of new data to correct the estimate is progressively weakened. Furthermore, PSIG-DP-TBD algorithm consistently shows superior performance compared to the MIG-DP-TBD algorithm, which corresponds approximately to the trajectory estimation results in Figure 10.

**Figure 14:**
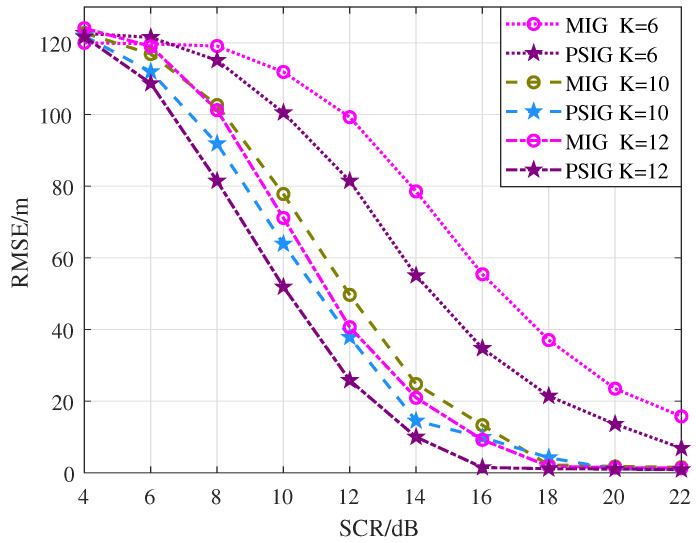
The root mean square errors of position for PSIG-DP-TBD and MIG-DP-TBD with different K.

In addition, the RMSE curves are given in Figure 14. The findings indicate that as the value of K increases, the precision of the estimated trajectory improves, demonstrating the effective integration of multi-frame information. Similarly, the RMSE of PSIG-DP-TBD is always lower than that of MIG-DP-TBD, implying better tracking accuracy.

To summarize, the synergistic advantages of the IG approach and the inter-frame integration method have been substantiated. The experimental findings indicate that the methods introduced here outperform traditional approaches in multi-frame detection scenarios. Furthermore, it is observed that the detection capabilities of these methods are augmented with an increase in the number of frames analyzed. It should be noted that increasing the frame numbers only limited performance improvements. The balance between performance enhancement and computational complexity must be carefully considered. Comparing the two proposed methods, the PSIG-DP-TBD outperforms MIG-DP-TBD in both detection and tracking performance. It demonstrates superior target trajectory estimation accuracy and detection robustness.

## 5. Conclusions

In this research, we have introduced an IG-based DP-TBD algorithm for range-azimuth measurements. By integrating a novel information geometry detector with a TBD algorithm, our approach effectively leverages inter-frame information to enhance the detection and tracking of weak moving targets in sea clutter backgrounds. Our method characterizes the trajectories of both the target and clutter on HPD or PS manifolds and designs an IG-based scoring function along the target’s motion trajectory. Utilizing dynamic programming and state transfer range, we have developed a robust solution model for target trajectory estimation and finally derived the IG-based DP-TBD algorithm for range-azimuth measurements. The proposed method has outperformed traditional approaches, yielding at least a 3 dB improvement in the SCR through experimental validation using real sea clutter data. This improvement underscores the algorithm’s effectiveness in addressing the challenges posed by weak target detection and tracking in complex clutter backgrounds.

Future research will take into account more complex target scenarios. It will progressively tackle challenges such as path crossing between multiple targets, and distinguishing adjacent multiple targets in complex backgrounds. Moreover, introducing machine learning methods [45,46], such as neural networks, into the multi-frame integration process to address the detection, tracking, and recognition of extended targets in radar systems is also an interesting research direction.

## Figures and Tables

**Table 1:** Radar parameters.

Parameters	Values
Operating frequency band	X
Operating frequency range	9.3∼9.5 GHz
Scanning bandwidth	25 MHz
Range resolution	6 m
Pulse repetition frequency	1.525 kHz, 3 kHz
Peak transmit power	50 W
Antenna length	1.8 m
Antenna operating mode	Circular scan
Antenna polarization method	HH
Antenna horizontal beamwidth	1.2°
Range sampling rate	60 MHz

## Data Availability

The data presented in this study are available at https://radars.ac.cn/web/data/getData?dataType=DatasetofRadarDetectingSea (accessed on 5 March 2025).
